# Double Barrel Nasal Trumpets to Prevent Upper Airway Obstruction after Nasal and Non-Nasal Surgery

**DOI:** 10.1155/2018/8567516

**Published:** 2018-03-20

**Authors:** Macario Camacho, Justin M. Wei, Lauren K. Reckley, Sungjin A. Song

**Affiliations:** Division of Otolaryngology, Sleep Surgery, and Sleep Medicine, Tripler Army Medical Center (Tripler AMC), 1 Jarrett White Rd., Honolulu, HI 96859, USA

## Abstract

**Objectives:**

During anesthesia emergence, patients are extubated and the upper airway can become vulnerable to obstruction. Nasal trumpets can help prevent obstruction. However, we have found no manuscript describing how to place nasal trumpets after nasal surgery (septoplasties or septorhinoplasties), likely because (1) the lack of space with nasal splints in place and (2) surgeons may fear that removing the trumpets could displace the splints. The objective of this manuscript is to describe how to place nasal trumpets even with nasal splints in place.

**Materials and Methods:**

The authors describe techniques (Double Barrel Technique and Modified Double Barrel Technique) that were developed over three years ago and have been used in patients with obstructive sleep apnea (OSA) and other patients who had collapsible or narrow upper airways (i.e., morbidly obese patients).

**Results:**

The technique described in the manuscript provides a method for placing one long and one short nasal trumpet in a manner that helps prevent postoperative upper airway obstruction. The modified version describes a method for placing nasal trumpets even when there are nasal splints in place. Over one-hundred patients have had nasal trumpets placed without postoperative oxygen desaturations.

**Conclusions:**

The Double Barrel Technique allows for a safe emergence from anesthesia in patients predisposed to upper airway obstruction (such as in obstructive sleep apnea and morbidly obese patients). To our knowledge, the Modified Double Barrel Technique is the first description for the use of nasal trumpets in patients who had nasal surgery and who have nasal splints in place.

## 1. Introduction

Some patients such as overweight or obese patients, obstructive sleep apnea (OSA) patients, and patients with craniofacial disorders are predisposed to upper airway obstruction. The act of changing from the upright to the supine position has been shown to reduce the upper airway volume by approximately 33% in a group of adult OSA patients [1]. Given that OSA patients are already predisposed to upper airway obstruction when they are in the supine position, adding the additional variable of general anesthesia can further predispose patients to upper airway obstruction during anesthesia induction and emergence. Additionally, there are other patients who may be predisposed to upper airway obstruction to include (1) morbidly obese patients, (2) patients with craniofacial deformities (i.e., mandibular insufficiency, retrognathia, and transverse maxillary deficiency), (3) patients with gigantism or acromegaly, (4) patients with Down syndrome, and (5) patients with significant oropharyngeal tissue hypertrophy (i.e., tonsillar and adenoid hypertrophy).

When a patient undergoes anesthesia induction, their upper airway is protected after the placement of an endotracheal tube. After surgery, when a patient is emerging from anesthesia, and is extubated, the upper airway can become vulnerable to obstruction. Over the years, several techniques to prevent upper airway obstruction after surgery have been evaluated. The use of nasal trumpets has been reported in several studies [2]. Normally if a patient has not had nasal surgery, then a nasal trumpet can be placed to help prevent nasal obstruction. Although the placement of a single nasal trumpet can help relieve obstruction, the upper airway can still collapse with a single nasal trumpet in place, especially if it is not long enough. Therefore, additional maneuvers are used to help open the upper airway (i.e., positive airway pressure and using a jaw thrust maneuver).

Thus far, however, we have found no manuscript describing how to place nasal trumpets after nasal surgery (septoplasties or septorhinoplasties) is performed, likely because (1) there is a lack of space because the nasal splints placed after nasal surgery crowd the nasal cavity and (2) surgeons may fear that removing the trumpets could displace the nasal splints that are meant to stay in for several days. The authors of this manuscript have developed a novel method for using nasal trumpets (Double Barrel Technique and Modified Double Barrel Technique) to help prevent upper airway obstruction after surgery even if the patients have had nasal surgery. This manuscript provides a technique used in our facility in over 100 patients to place nasal trumpets even with nasal splints in place. The objective of this is to describe two techniques which have helped prevent upper airway obstruction after anesthesia, the first describing the use of two different length nasal trumpets in patients who have not had nasal surgery and the modification of the technique which allows placement of the nasal trumpets even in patients who had nasal surgery and have nasal splints in place.

## 2. Materials and Methods

### 2.1. Compliance with Ethical Standards

As a techniques paper, this manuscript is not research and therefore does not require review by the Investigational Review Board (IRB) at our institution. There was no retrospective chart review performed. The project does not meet the Federal definition of research (DHHS 45 CFR 46.102(d)) or the Federal definition of clinical investigation (FDA 21 CFR 50.3(c) and 56.102(c)). This article does not contain any studies with human participants performed by any of the authors.

### 2.2. Methodology for Developing the Techniques

The first step was to search the literature to evaluate techniques for stenting the upper airway open to prevent upper airway obstruction on emergence from anesthesia, especially in patients who have undergone nasal surgery and have nasal splints in place. If there was no manuscript describing a technique, then the plan was to use innovative brainstorming and logical reasoning with regards to how nasal trumpets could be placed into patients who have nasal splints already in the nasal cavity. Lastly, if no techniques were found, then the plan was to describe the technique we developed.

### 2.3. Search Strategy

In order to identify if there were any studies describing the use of nasal trumpets during emergence of anesthesia in patients who have had nasal surgery, we used the keywords, terms, and phrases in combinations as follows: “nasal stent,” “nasal trumpet,” “nasopharyngeal trumpet,” “nasopharyngeal airway stent,” “nasopharyngeal airway tube,” “nasopharyngeal obturator,” or “nasopharyngeal tube” in conjunction with and without the keyword “anesthesia.” The search strategy for PubMed/MEDLINE is as follows: ((“nasal stent” OR “nasal trumpet” OR “nasopharyngeal trumpet” OR “nasopharyngeal airway stent” OR “nasopharyngeal airway tube” OR “nasopharyngeal obturator” OR “nasopharyngeal tube”) AND (anesthesia)).

## 3. Results

### 3.1. Literature Search

A review of the literature through December 10, 2017, identified forty articles [3–42] discussing the use of nasal devices and sleep disordered breathing, with the majority discussing the use in prevention of upper airway obstruction. Of the published manuscripts, the far majority that quantified the use of upper airway obstruction used nasal trumpets. Kumar et al. performed a meta-analysis and reported a 57% reduction in the apnea-hypopnea index in patients who were measured and the mean increase of the lowest oxygen saturation from 66.5% to 75.5% [2].

No manuscript was identified for describing how to place nasal trumpets after nasal surgery (septoplasties or septorhinoplasties).

### 3.2. Technique Development

Because there were no published studies on the topic, we reasoned logically using our knowledge of the structures of the upper airway that could obstruct during anesthesia emergence, and we examined the various nasal trumpet sizes and types and evaluated how to properly position nasal trumpets against nasal splints in a manner that they could both fit into the nasal cavity. After a thorough evaluation, it was determined that cutting the medial aspect of the outer flange of the nasal trumpets would allow for the nasal trumpet to rest fully against the nasal splint. Therefore, by being flush against each other, the nasal trumpet and the nasal splint were essentially one unit with regard to being placed into the nasal cavity and upper airway. Given that the nasal trumpets and nasal splints are two separate units, adding lubrication in the form of mupirocin allows for them to slide easily against each other with minimal friction, and thus the nasal trumpets could be removed from the nasal cavity without dislocating or disrupting the position of the nasal splints. The authors have used the technique for over three years, in over one-hundred patients with OSA and other patients who had collapsible or narrow upper airways (i.e., morbidly obese patients), with no oxygen desaturations. The techniques are called (1) The Double Barrel Technique for patients who have not had nasal surgery and (2) The Modified Double Barrel Technique for patients who have had nasal surgery and have nasal splints in place.

### 3.3. Technique Descriptions

#### 3.3.1. Technique for Non-Nasal Surgery Patients (Double Barrel Technique)

**Figure 1:**
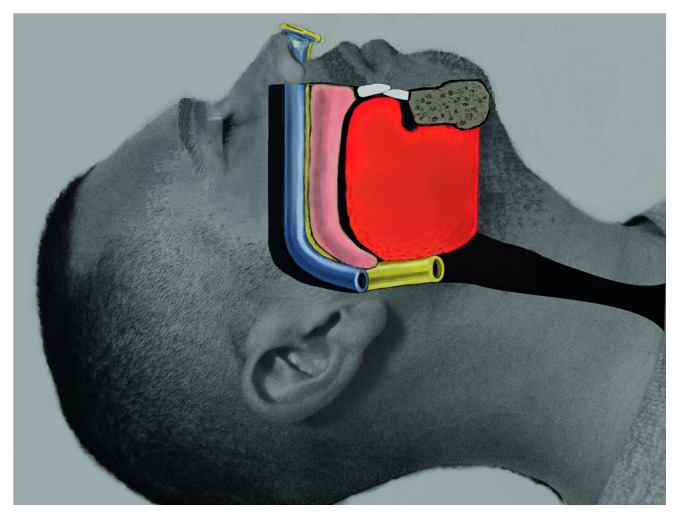
The Double Barrel Technique. The image demonstrates the technique, with two nasal trumpets in the upper airway. The left nasal cavity and upper airway has a longer nasal trumpet (yellow) and the right nasal cavity and upper airway has a shorter nasal trumpet (blue). The combination of the two nasal trumpets provides a physical method for stenting the upper airway open. Note: the image is a modified version of a contribution that the first author of this manuscript (Macario Camacho) made to Wikimedia Commons (open source images).

For the Double Barrel Technique, two nasal trumpets are placed (one longer nasal trumpet and one shorter nasal trumpet) before the patient is extubated. By having a longer nasal trumpet that extends farther (to the base of tongue or hypopharynx) and a shorter nasal trumpet that extends just beyond the soft palate, the patients will have a stented upper airway pathway for air to flow (Figure 1). The steps for the Double Barrel Technique include (1) decongest the nose with oxymetazoline (if the patient did not undergo inferior turbinoplasty surgery), (2) place a layer of mupirocin ointment on the nasal trumpets, (3) place the longer nasal trumpet into the nostril in which there is more space available based on inferior turbinate size and presence of a nasal septal deviation, (4) place the smaller nasal trumpet into the nostril in which there is less space available, and (5) keep the nasal trumpets in place until the patient is in the postanesthesia care unit (PACU) and is alert enough to request the removal of the nasal trumpets, at which point they can be removed by sliding them out.

#### 3.3.2. Technique for Nasal Surgery Patients with Nasal Splints in Place (Modified Double Barrel Technique)

**Figure 2:**
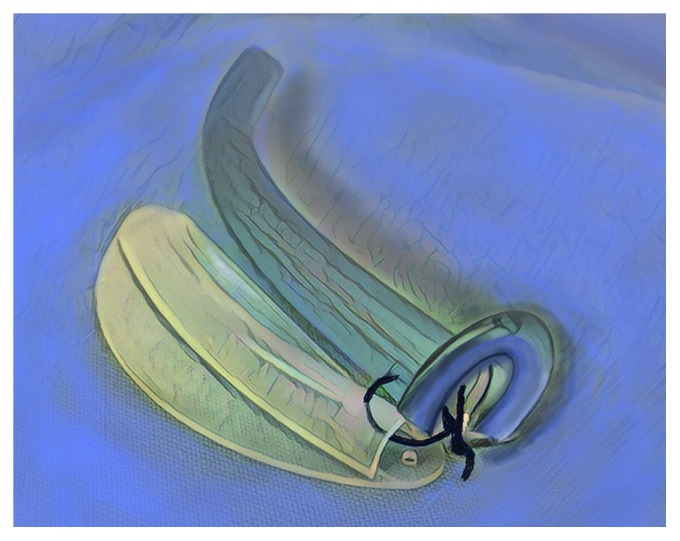
The Modified Double Barrel Technique. This combination of nasal trumpets and nasal splints allows for simultaneous placement of nasal trumpets in the presence of nasal splints. Trim away approximately one-third of the medial aspect of the outer flange of the nasal trumpets and use black suture to keep them together. Slide in the nasal trumpets and the nasal splints into the nasal cavity as a unit (starting with the tip of the nasal trumpets and then insert the nasal splints together with the trumpets as a unit). Note: make sure to place one longer nasal trumpet on one side and one shorter nasal trumpet on the opposite side.

If the patients just have had nasal surgery and have nasal splints sutured in place, then the nasal trumpets are placed lateral and inferior to the nasal splints using the Modified Double Barrel Technique, see Figure 2 for setup. For the Modified Double Barrel Technique, (1) decongest the nose with oxymetazoline (if the patient did not undergo inferior turbinoplasty surgery), (2) trim away approximately one-third of the medial aspect of outer flange of the nasal trumpets so that they can rest against the nasal splints (Figure 2), (3) use black nylon or black silk suture to keep the nasal trumpets attached to the nasal splints, so they sit in the proper position, (4) place a layer of mupirocin ointment on the nasal splints and on the nasal trumpets, (5) slide in the nasal trumpets and the nasal splints as a unit (starting with the tip of the nasal trumpets and then insert the nasal splints together with the trumpets as a unit) (note: make sure to place one longer nasal trumpet on one side and one shorter nasal trumpet on the opposite side), (6) use blue prolene suture as the trans-septal suture for securing the nasal splints in the standard fashion, and (7) the black nylon or black silk suture is cut and the nasal trumpets are removed after the patient is alert enough in PACU to request removal. Because the only suture holding the nasal trumpets to the nasal splints is the black suture, once the black suture is cut, the nasal trumpets slide out fairly easily as they are independent and mobile.

### 3.4. Description of Nasal Trumpet Size Combinations

Given that the nasal trumpets are different lengths and sizes, various combinations have been used at our institution. In general, one longer nasal trumpet and one shorter nasal trumpet are used in combination, but given that patients' soft palates are different lengths and the distance to their hypopharynx are different lengths, Table 1 lists the nasal trumpet combinations that are typically used at our institution. In general, a smaller woman will benefit from shorter nasal trumpets (i.e., 26–28 French) and large men will benefit from longer nasal trumpets (i.e., 30–34 French).

## 4. Discussion

Overall, we are excited to present the techniques from this manuscript as they have improved postanesthesia oxygen saturations, especially in OSA and morbidly obese patients. The techniques are easy to follow. First and foremost, this paper describes a technique that to our knowledge has not been described in the literature before, which is that it describes how to place nasal trumpets in patients who have undergone nasal surgery and have nasal splints in place. Nasal trumpets have been used safely as a method to help prevent upper airway obstruction on emergence from anesthesia. However, the major challenge prior to this manuscript is that there is no publication describing any method to place nasal trumpets in patients who have had nasal surgery. At first, we only used one nasal trumpet, but noticed that some patients still had desaturations. Then we used two nasal trumpets. Initially, nasal trumpets of same lengths were used; however, after logical reasoning, the authors came to the realization that having one shorter and one longer nasal trumpet would actually stent the airway much better. It makes sense that if two long nasal trumpets are used, then it is possible that the trumpets could be sitting in the piriform sinuses or at the base of tongue and therefore, tongue could still obstruct the airflow and prevent proper ventilation. If two short nasal trumpets are used and they reach just beyond the soft palate, the tongue could still obstruct the upper airway and negatively affect proper ventilation below the level of the nasal trumpets. However, as described in the Double Barrel Technique if you place one longer trumpet (extending to the piriform sinuses or to the level of the base of tongue) and you place one shorter nasal trumpet that extends just beyond the soft palate, then the combination of the two is very likely to stent the upper airway open (Figure 1).

Second, the Double Barrel Technique and Modified Double Barrel Technique have both allowed safe emergence from anesthesia for patients predisposed to upper airway obstruction. The nasal trumpets described herein have been used safely in our institution for over three years without any patient having complications or desaturations. The Double Barrel Technique has been embraced by the anesthesiology providers for non-head and neck procedures as well since it allows for unobstructed airflow on emergence. For patients who have had nasal surgery and the Modified Double Barrel Technique is used, the color coding for the suture also makes it easy to tell the difference between the suture to be cut in the PACU (black for the nasal trumpet suture) and the suture to be left intact (blue for the nasal splint suture) after nasal surgery. We have also provided training to the nurses in the PACU on the proper removal of nasal trumpets and the far majority of them are comfortable cutting the black suture and removing the nasal trumpets on their own once the patients are fully awake and ask for them to be removed. Additionally, the splints have remained in place after removal of the nasal trumpets in all cases without displacement of the nasal splints. However, although we have not had any displacement of the nasal splints, it is possible that if not enough lubrication is placed between the nasal splints and nasal trumpets or if the trumpets are removed too aggressively, then they could be shifted or displaced.

Lastly, although the nasal passages are not the cause of upper airway obstruction in most cases, they are important in the perioperative management of a patent upper airway, especially during induction and emergence from anesthesia. The use of nasal trumpets perioperatively in patients who are predisposed to upper airway obstruction can be an important adjunct in the maintenance of a patent upper airway. The nasal cavity is a generally rigid structure made up of bone, cartilage, and nonfloppy, erectile soft tissues (i.e., inferior turbinates which can be small, medium, large, and very large (grades 1 to 4)) [43]. Nasal breathing is generally the preferred breathing modality, and nasal patency is important in providing positive airway pressure via mask anesthesia. The use of nasal trumpets after surgery provides a fixed and rigid method for maintaining the upper airway patent as the devices literally stent the upper airway open, from the nasopharynx down to the hypopharynx. However, as this manuscript has described previously, having nasal surgery generally precludes patients from being able to have nasal trumpets placed. Herein, the Double Barrel and Modified Double Barrel techniques allow for placement of nasal trumpets bilaterally in patients who have OSA, are obese, have craniofacial syndromes, or are otherwise predisposed to upper airway obstruction.

## 5. Conclusions

The Double Barrel Technique allows for a safe emergence from anesthesia in patients predisposed to upper airway obstruction (such as in obstructive sleep apnea and morbidly obese patients). To our knowledge, the Modified Double Barrel Technique is the first description for the use of nasal trumpets in patients who had nasal surgery and have nasal splints in place.

## Figures and Tables

**Table 1:** Typical nasal trumpet sizes and combinations used based on patient body sizes.

Patient sizes	Typical nasal trumpet sizes (Fr)
Large man	34, 30
Medium man	32, 28
Small man	30, 28
Large woman	32, 28
Medium woman	30, 28
Small woman	28, 26

Note: this is a general guide; however, the nasal trumpet sizes are also limited by additional physical attributes such as the presence of nasal septal deviations, the sizes of the inferior turbinates, and the general upper airway anatomy. Fr = French.
